# The effect of subcutaneous and intraperitoneal anesthesia on post laparoscopic pain: a randomized controlled trial

**DOI:** 10.1038/s41598-020-80130-6

**Published:** 2021-01-08

**Authors:** Ohad Gluck, Elad Barber, Ohad Feldstein, Ori Tal, Ram Kerner, Ran Keidar, Inna Wolfson, Shimon Ginath, Jacob Bar, Ron Sagiv

**Affiliations:** Departments of Obstetrics and Gynecology, The Edith Wolfson Medical Center, Holon, and Sackler School of Medicine, Tel Aviv University, P.O. Box 5, 58100 Holon, Israel

**Keywords:** Clinical trial design, Randomized controlled trials, Medical research, Outcomes research, Pain

## Abstract

A few modes of perioperative local analgesia have been studied in order to reduce postoperative pain after laparoscopy, including preemptive local anesthetics in the trocar sites and intraperitoneal anesthetics administration at the end of the surgery. However, the evidence regarding their efficacy are conflicting. In addition, the combination of both aforementioned methods has been rarely studied. Our aim was to evaluate whether subcutaneous trocar site and/or intraperitoneal analgesia reduce pain after gynecologic operative laparoscopy. This was a single-centered, randomized, controlled, double-blinded trial. The patients were randomly assigned to one of four equally sized groups: group 1—subcutaneous and intraperitoneal analgesia; group 2—subcutaneous analgesia and intraperitoneal placebo; group 3—subcutaneous placebo and intraperitoneal analgesia; Group 4—subcutaneous and intraperitoneal placebo. The patients, the surgeons, and the pain evaluators were all blinded to the patient’s allocation. Included were patients who underwent elective operative laparoscopy. Exclusion criteria were: active infection, pregnancy, known sensitivity to Bupivacaine-Hydrochloride, chronic pelvic pain, surgeries with additional vaginal procedures, conversion to laparotomy, and malignancy. A total of 9 ml of Bupivacaine-Hydrochloride (Marcaine) 0.5%, or Sodium-Chloride 0.9%, as a placebo, were injected subcutaneously to the trocar sites (3 ml to each trocar site), prior to skin incision. In addition, 10 ml of Bupivacaine-Hydrochloride 0.5%, diluted with 40 ml of Sodium-Chloride 0.9% (a total of 50 ml solution), or 50 ml of Sodium-Chloride 0.9%, as a placebo, were injected intraperitoneally at the end of the surgery. By utilizing the 10 cm Visual-analogue-scale (VAS) we assessed post-operative pain at rest at 3, 8, and 24 h, and during ambulation at 8 and 24 h. The study was approved by the local Institutional Review Board and has been registered at clinicaltrials.gov. We conformed to the CONSORT recommendations. Between December 2016 and July 2019, a total of 119 patients were included in the study. Demographic and interventional characteristics were similar among the groups. The level of postoperative pain, either at rest or with change of position, was not significantly different between the groups, at all-time points. Application of subcutaneous and/or intraperitoneal analgesia is not effective in reducing pain after gynecologic operative laparoscopy.

Clinical trial identification number: NCT02976571. Date of trial registration 11/29/2016. URL of the registration site: https://clinicaltrials.gov.

## Introduction

One of the techniques for reducing pain and opiate demand after surgery is the use of local anesthesia in surgical incisions^1^. It has been shown that nociceptive stimuli can alter the electrophysiological processes in the neurons^2^.This alteration results in a lower pain threshold and an increased response to pain stimuli.

By infiltrating a local anesthetic before the incision is made, these effects should in theory be avoided. This, in turn, can potentially increase mobilization and shorten hospitalization after surgery^1^.

In an attempt to reduce postoperative pain following laparoscopy, a variety of methods of perioperative local analgesia have been studied. Injection of preemptive local anesthetics into the trocar sites has been shown to be beneficial in reducing postoperative pain during ambulation^3^, whereas administration of intraperitoneal anesthetics at the end of surgery was found to be effective in reducing intensity of postoperative abdominal pain^4,5^, as well as shoulder-tip pain^6^. However, a few studies failed to show these beneficial effects of both trocar site local anesthesia^7^, and the intraperitoneal anesthesia^8,9^. A recent systematic review by Long et al.^10^ examined the evidence regarding the practice of preemptive analgesia administration; they reported that laparoscopic incisional infiltration has a modest effect, although data regarding this intervention are inconclusive. In addition, intraperitoneal analgesia was reported to likely be beneficial for postoperative pain control^10^. Nevertheless, the combination of incisional site and intraperitoneal analgesia has rarely been studied, and the evidence is conflicting^11–14^.

Therefore, our goal was to study how administration of preemptive incisional site subcutaneous (SC) anesthetics, combined with-operative intraperitoneal (IP) analgesia, affects postoperative pain levels (abdominal and shoulder-tip) among patients undergoing operative gynecologic laparoscopy.

## Results

**Figure 1:**
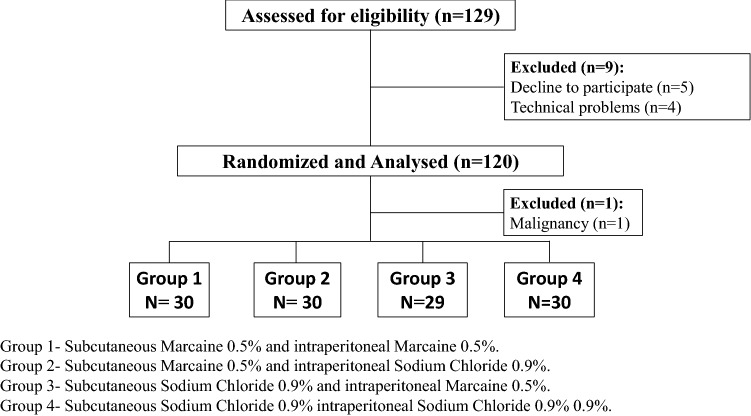
Patients enrolment.

During the study period, 129 patients met the inclusion criteria. Of these, five patients declined to participate, and additional four patients were excluded due to technical reasons (none of the investigating surgeons was available). After enrollment, one patient was found to have malignancy during the operation. Ultimately, 119 patients (group 1—30, group 2—30, group 3—29, group 4—30) completed full pain assessment (Fig. 1). All patients returned for a follow up visit. No early / late complications or adverse effects were registered for the study population. No malignancy was detected on final pathological examination (for the patient who completed enrollment and were included in the final analysis).

We did not detect any differences between the study groups and the patient and surgery characteristics (Table 1). Also, there was no difference in the frequency of major vs. minor surgeries between the study groups Tables (2, 3).

**Table 1:** Comparison of women and surgery characteristics between the study groups.

Variable name	Group1 N = 30	Group2 N = 30	Group3 N = 29	Group4 N = 30	*p* Value
Age mean ± S.D	45.1 ± 11.7	43.8 ± 18.2	44.9 ± 13.9	46.4 ± 14.5	*p* = 0.929
BMI mean ± S.D	25.6 ± 4.5	26 ± 5.3	25.5 ± 2.9	26.5 ± 2.9	*p* = 0.748
Previous surgeries N;%	1.7 ± 0.5	1.4 ± 0.6	1.4 ± 0.8	1.4 ± 0.5	*p* = 0.432
Menopause N;%	8 (27%)	8 (27%)	7 (24%)	7 (23%)	*p* = 0.901
Surgery length (minutes) mean ± S.D	71.2 ± 36.5	64.3 ± 34.2	73.6 ± 44.6	70.9 ± 45.3	*p* = 0.830
Blood loss mean ± S.D	66.7 ± 99.9	92 ± 105.7	44.6 ± 81.9	86.3 ± 108.2	*p* = 0.266
Gravity mean ± S.D	2.1 ± 1.9	1.4 ± 1.9	2.3 ± 1.8	2.4 ± 3.0	*p* = 0.259
Parity mean ± S.D	1.5 ± 1.1	1.1 ± 1.8	1.5 ± 1.3	1.2 ± 1.3	*p* = 0.657

Group 1—Subcutaneous drug and intraperitoneal drug.

Group 2—Subcutaneous drug and intraperitoneal placebo.

Group 3—Subcutaneous placebo and intraperitoneal drug.

Group 4—Subcutaneous placebo and intraperitoneal placebo.

**Table 2:** Surgery type according to study group.

	Group 1 30	Group 2 30	Group 3 29	Group 4 30
BSO	8 (27%)	9 (30%)	9 (31%)	11 (37%)
Cystectomy	5 (17%)	11 (37%)	6 (20%)	10 (33%)
Endometrioma resection	1 (3%)	1 (3%)	0 (0%)	0 (0%)
Hysterectomy	7 (23%)	3 (10%)	8 (27%)	4 (13%)
Myomectomy	0 (0%)	0 (0%)	1 (3%)	1 (3%)
Salpingectomy	2 (7%)	2 (7%)	1 (3%)	1 (3%)
USO	7 (23%)	4 (13%)	3 (10%)	1 (3%)

BSO: Bilateral salpingo-oophorectomy.

USO: Unilateral salpingo-oophorectomy.

**Table 3:** Comparison between major and minor surgeries according to study groups*.

	Group 1 30	Group 2 30	Group 3 29	Group 4 30
Minor*	22 (73%)	26 (87%)	21 (72%)	25 (83%)
Major**	8 (27%)	4 (13%)	9 (28%)	5 (17%)

*Minor surgeries: Cystectomy, Salpingectomy, Unilateral/Bilateral salpingo-oophorectomy.

**Major surgeries: Hysterectomy, Myomectomy, Endometrioma resection.

*p Value = 0.34.

No significant associations were found between the study groups and the discrete visual analog scale (VAS) scores in 3, 8 and 24 h after surgery, in either univariate or multivariate analyses (Table 4).

**Table 4:** Univariate comparison of post-operative pain assessment and analgesic requirements according to study groups.

Variable name	Group 1 N = 30	Group 2 N = 30	Group 3 N = 29	Group 4 N = 30	*p* Value
Analgesics requirements during recovery					
MME mean ± SD	14.1 ± 7.3	12.0 ± 7.0	13.2 ± 7.9	12.1 ± 7.8	*p* = 0.665
Pain assessment and analgesics requirements at 3 h after surgery					
Shoulder pain at rest mean ± SD	1.3 ± 2.7	0.9 ± 2.5	0.7 ± 2.4	0.9 ± 2.0	*p* = 0.827
Abdominal pain at rest mean ± SD	4.8 ± 2.7	3.7 ± 2.9	4.0 ± 3.0	4.4 ± 2.5	*p* = 0.496
Analgesia—all N;%	9 (30%)	5 (17%)	9 (31%)	11 (37%)	*p* = 0.374
Pain assessment and analgesics requirements at 8 h after surgery					
Shoulder pain at rest mean ± SD	2.0 ± 2.9	1.4 ± 2.5	0.7 ± 1.7	1.4 ± 2.5	*p* = 0.267
Abdominal pain at rest mean ± SD	3.0 ± 3.0	3.7 ± 2.8	3.1 ± 2.8	4.0 ± 2.6	*p* = 0.441
Abdominal pain at movement mean ± SD	4.0 ± 3.3	4.3 ± 3.0	4.6 ± 3.1	5.0 ± 3.1	*p* = 0.678
Analgesia—all N;%	15 (50%)	10 (33%)	14 (48%)	14 (46%)	*p* = 0.574
Pain assessment and analgesics requirements at 24 h after surgery					
Shoulder pain at rest mean ± SD	1.8 ± 2.6	1.4 ± 2.6	1.2 ± 2.4	1.8 ± 2.8	*p* = 0.753
Abdominal pain at rest mean ± SD	2.9 ± 2.4	3.7 ± 2.4	3.0 ± 3.0	4.2 ± 3.0	*p* = 0.249
Abdominal pain at movement mean ± SD	8.6 ± 5.6	9.6 ± 6.1	8.4 ± 5.6	10.4 ± 7.5	*p* = 0.566
Analgesia—all N;%	12 (40%)	11 (37%)	14 (47%)	7 (23%)	*p* = 0.292

*Comparisons of longitudinal data (MME, VAS scores) were performed using One Way Analysis of variance (ANOVA). Comparison of Dichotomous variables (Analgesic treatment and treatment with opiates at 3, 8 and 24 h post surgery) were performed using the Chi-square or Fisher exact test.

**MME—Morphine Milligram Equivalents; VAS—visual analog scale.

No significant association was found between the study groups and the Morphine Milligram Equivalents (MME) given during recovery (Table 4). Only 6 patients were treated with opioids 3 h post-surgery (there was no difference between the groups). None needed such treatment at and 8 and 24 h post-surgery. Furthermore, we did not detect differences in the risk for pain that required analgesic treatment in both univariate and multivariate analyses (Table 5).

**Table 5:** Crude and adjusted odds ratios for analgesic treatment using logistic mixed effects models*.

Variable	Group	Crude OR	95%CI	Adjusted OR	95%CI
Analgesic requirement at 3 h after surgery					
Analgesia (Yes/No)	Reference	1.0		1.0	
	Group2	0.95	0.31 to 2.89	0.95	0.31 to 2.92
	Group3	1.29	0.44 to 3.80	1.06	0.35 to 3.26
	Group4	0.46	0.13 to 1.61	0.50	0.14 to 1.75
Analgesic requirement at 8 h after surgery					
Analgesia (Yes/No)	Reference	1.0		1.0	
	Group2	1.07	0.39 to 2.97	0.99	0.35 to 2.79
	Group3	0.94	0.34 to 2.61	1.11	0.39 to 3.15
	Group4	0.56	0.20 to 1.62	0.57	0.20 to 1.68
Analgesic requirement at 24 h after surgery					
Analgesia (Yes/No)	Reference	1.0		1.0	
	Group2	0.71	0.25 to 2.02	0.73	0.26 to 2.11
	Group3	0.33	0.11 to 1.01	0.35	0.11 to 1.10
	Group4	0.65	0.23 to 1.86	0.75	0.26 to 2.19

*The univariate and multivariate models were fitted using the surgeon’s identity as a random effect. The type of surgery (major vs. minor) and the Morphine Milligram Equivalents (MME) given during recovery were used as fixed effects in the multivariate models.

When comparing patients who were treated with and without subcutaneous anesthesia and patients with and without intra-peritoneal anesthesia, we did not detect differences in both pain assessment and analgesic prescription outcomes.

## Discussion

We did not detect any difference among the study groups in postoperative levels of pain with change of position or at rest, or analgesic use, at any point in time, in both univariate and multivariate analyses. Also, differences were not found when comparing patients who were treated with and without subcutaneous anesthesia and patients with and without intra-peritoneal anesthesia.

Ghezzi et al.^15^ and Grube et al.^16^ also failed to show effectiveness of preemptive trocar-site analgesia in reducing postoperative pain. Arden et al.^17^ reported that intraperitoneal instillation of bupivacaine at the end of laparoscopic hysterectomy did not reduce postoperative pain, nor did it affect the utilization of opioid analgesics.

Nevertheless, a few studies reported beneficial effect for the aforementioned interventions. Ravndal et al.^3^ reported that preemptive local anesthetics injected into the trocar sites reduced postoperative pain at ambulation 5 h after surgery. Chou et al.^4^ showed that the combination of preoperative and postoperative intraperitoneal Bupivacaine reduced pain at 2 and 4 h postoperatively.

A few systematic reviews have also addressed the role of different interventions for reducing postoperative pain. Ong et al.^18^ found no beneficial effect of preemptive local anesthetic wound infiltration on postoperative pain; however, this intervention was shown to reduce postoperative analgesic consumption. Marks et al.^5^ found that analgesia instilled intraperitoneally significantly decreased pain during a 6-h interval after laparoscopy. Long et al.^10^ studied the evidence regarding the practice of preemptive analgesia in various forms. They reported modest effect of incisional infiltrations and stated that conclusions drawn by previous studies are conflicting. In addition, they found that intraperitoneal analgesia given upon completion of surgery is likely beneficial.

Our findings, namely the lack of effectiveness for both incisional site and intraperitoneal analgesia administration, could have a number of explanations. First, it is possible that the analgesic dosage we used is insufficient. The maximal dosage of Bupivacaine allowed for local analgesia in adults is 175 mg, since it is associated with the highest risk of cardiovascular toxicity among the various local anesthetics available^19^. Because we combined two modalities of analgesia (subcutaneous and intraperitoneal), we chose the lowest dose that had been shown to be efficient for each modality^20^. Therefore, it is possible that due to safety concerns we failed to reach the threshold for adequate postoperative pain relief. Secondly, most patients in our study underwent minor surgeries, such as diagnostic laparoscopies and salpingectomies. It is possible that if we included only major surgeries (such as hysterectomies and myomectomies), higher levels of pain would have been reported and statistically significant differences in primary and/ or secondary outcomes would have been obtained. Nevertheless, it is important to mention that 5 mg (20 ml of 0.25%) Bupivacaine instilled subcutaneously was reported to be effective in reducing postoperative pain even in diagnostic laparoscopies^21^. It is also possible that the immediate postoperative pain relief, was a confounding factor since it lowered the level of postoperative pain. However, as all the medications administered at recovery room have an elimination half-life (T1/2) of up to six hours, together with the fact that no difference in MME was detected between the groups, it is safe to assume that the immediate postoperative pain relief did not affect the primary outcome. Lastly, our patients possibly reported only low to medium postoperative pain because they were merely asked to move from prone to sitting position, rather than engage in more strenuous activities, such as walking in the ward.

This study has several strengths. To the best of our knowledge, this is the first study that compared 4 types of interventions—SC analgesia only, IP analgesia only, combined SC and IP analgesia, and no analgesia at all (placebo). Moreover, the randomized, double-blinded design, as well as implementing standardized intraoperative and postoperative protocols for pain relief, has minimized the risk for potential bias. Lastly, we investigated the effect of preemptive analgesia on postoperative levels of pain and utilization of medication, including the demand for opioid-based analgesics.

That said, our study is not free of limitations. To begin with, we only assessed short-term outcomes, namely the first 24 h following surgery, and did not evaluate the full recovery period which culminates with the patient returning to full activity. However, as no differences were detected in the first 24 h, it is unlikely we would have found any differences in later periods. Another setback is that we did not evaluate the effect of the various interventions on the time elapsed until full mobility. Yet, it is important to emphasize that no thromboembolic events, which are a major concern in non-mobile patients, were recorded. Lastly, the surgeries included in this study vary in baseline levels of pain expected, and this variation may be a confounding factor. However, as mentioned earlier, the groups were not significantly different as for the frequency of major or minor surgeries, so it is safe to assume that this had a negligible effect on the results, if at all.

In conclusion, it appears that administration of preemptive subcutaneous trocar-site analgesia with or without administration of intraperitoneal analgesia upon completion of surgery does not affect the level of postoperative pain.

## Methods

This was a 4-arms randomized controlled trial. Participants were recruited from the Gynecologic Department at the Edith Wolfson Medical Center, Holon, Israel, from December 1st, 2016 until July 31st, 2019. Inclusion criteria included patients with good general health (defined as American Society of Anesthesiologists grade 1–2), who were undergoing a laparoscopic surgery for benign indications. Exclusion criteria included patients who suffered from chronic pelvic pain (non-menstrual pain of 6 or more months, that alters daily function or necessitates medical or surgical treatment), pregnancy, allergy to Bupivacaine hydrochloride, emergency surgeries, execution of additional vaginal procedures, and surgeries performed for the following indications: active pelvic infection, ectopic pregnancy, or malignancy. In order to eliminate possible confounders that might increase the level of postoperative pain independently, post-assignment exclusion was executed in cases of conversion to laparotomy and intraoperative diagnosis of malignancy (confirmed by frozen section pathology).

This study was approved by the Wolfson medical center review board (approval number 0198-16-WOMC, dated 03/11/2016), and written informed consent was obtained from all subjects participating in the trial. The trial was registered prior to patient enrollment at clinicaltrials.gov (NCT02976571, Principal investigator Ohad Gluck, date of trial registration 11/29/2016). The clinical trial was registered prior to patient enrollment.

This manuscript adheres to the applicable CONSORT guidelines^22^.

This is a double-blinded study, with parallel assignments, to one of the four following groups:

- Group 1—SC Bupivacaine hydrochloride (AstraZeneca, Sodertalje, Sweden) 0.5% and IP Bupivacaine hydrochloride 0.5%.
- Group 2—SC Bupivacaine hydrochloride 0.5% and IP Sodium Chloride 0.9% (NaCl).
- Group 3—SC NaCl 0.9% and IP Bupivacaine hydrochloride 0.5%.
- Group 4—SC NaCl 0.9% and IP NaCl 0.9%.

After providing written informed consent, patients were randomly assigned to one of the study groups.

The randomization was performed in blocks of 40, using an on-line randomization program (http://www.randomization.com). In order to ensure allocation concealment the random allocation sequence was kept with the lead author (OG), which was not a part of the recruiting, operating, or pain assessment team.

An assigned nurse (IW) prepared syringes prior to each surgery, outside of the operation room, according to the randomization list, and unlabeled syringes were provided to the surgeons upon commencing surgery. The content of each syringe was written in the concealed list only. Patients, surgeons, anesthesiologists, and pain assessment researchers were all blinded to patients’ allocations.

Syringes were prepared according to the following instructions:

The first syringe, for SC injection, contained 9 ml of Bupivacaine hydrochloride 0.5% or NaCl 0.9%, as placebo. Three ml of this solution were injected subcutaneously (exclusively beneath the skin) at each trocar site, prior to skin incision.

The second syringe, for IP instillation, contained 10 ml of Bupivacaine hydrochloride 0.5% or 10 ml of NaCl 0.9%, as placebo, with an additional 40 ml of NaCl 0.9% (50 ml total volume). This solution was instilled intra-abdominally prior to abdominal closure by irrigating the diaphragm and the pelvis (15 ml under each diaphragm, and 20 ml in the pelvis). All surgeons administered the solutions at the same locations and in the same manner.

In order to study the short-term and long-term effects of intervention, postoperative pain at rest was evaluated at 3, 8, and 24 h after surgery. According to our protocol, patient ambulation starts 8 h following surgery; therefore, postoperative pain with change of position was evaluated at 8 and 24 h after surgery.

Prior studies have utilized the VAS for evaluating similar outcomes^12,21,23^. Therefore, we used a 10-cm VAS to scale the level of pain. For assessing pain at rest, prone patients were asked to grade the level of pain in the abdomen and at the tip of the shoulder on a scale of 0 (defined as no pain) to 10 (defined as the worst pain ever experienced). For assessing pain with change of position, patients were asked to grade their pain using the same scale, while shifting from lying in bed to sitting in a chair. A member of the surgical team who was not involved in the index surgical case administered the VAS scale.

We conducted a preoperative fasting of 6 h for solids and liquids, and formulated a standardized protocol for preoperative management, anesthesia, and analgesia.

All patients received general anesthetic induction and maintenance. Patients were premedicated with 2 mg intravenous (IV) Midazolam (Rafa, Jerusalem, Israel). General anesthesia was induced with 2 mg/kg IV Propofol (Fresenius Kabi, Graz, Austria), 2 mg/kg IV Meperidine (Panpharma, Luitre, France), and 0.6 mg/kg IV Rocuronium Bromide (Unipharm, Melsungen, Germany). General anesthesia was maintained with an infusion of 50–150 mg/kg/min IV Propofol, combined with inhaled Sevoflurane (LGM pharma, USA) and oxygen. Under general anesthesia with endotracheal intubation, the patient was positioned properly and draped. Antibiotics and thrombophylaxis were given when indicated.

In the recovery ward, pain medication was provided by the attending nurses upon demand, according to the following regime:

- First-line: 1 g IV Paracetamol, up to 4 times per day upon demand.
- Second-line: 1 g oral Dipyrone (not available in the USA), up to 4 times per day upon demand.
- Third-line: Opioid-based medication, either 2–3 mg IV Morphine (As Kalceks, Riga, Latvia) or 20–30 mg IV Meperidine.

Upon transfer to the postoperative ward, a uniform, standardized, postoperative pain-relief regime was applied for all patients, consisting of:

- First-line: 1 g IV Paracetamol, up to 4 times per day upon demand.
- Second-line: 1 g oral Dipyrone, up to 4 times per day upon demand.
- Third-line: 75 mg Intramuscular Diclofenac Sodium (Teva, Petah-Tikva, Israel) 75 mg, up to 3 times per day upon demand.
- Fourth-line: 100 mg IV Tramadol Hydrochloride (Dexcel, Or-Yehuda, Israel), up to 3 times per day upon demand, usually along with 10 mg IV Metoclopramide (Rafa, Jerusalem, Israel).

All procedures were performed by one of three senior surgeons in our department. Three trocars were placed in all laparoscopies—umbilical (5-mm), another 5-mm trocar, positioned at the left lower abdomen, and an additional 10-mm trocar placed in the supra-pubic area. Both SC and IP solution administration was performed in the same manner by all surgeons, as described above. At the end of each surgery, the fascia was closed using absorbable suture (Vicryl 1, Ethicon), and the skin was closed using skin glue (Dermabond, Johnson and Johnson).

All but 8 patients were discharged on postoperative day 1, according to our departmental protocol. The latter requested later discharge and therefore discharged on postoperative day 2–3. The patients were instructed to use non-opioid, over-the-counter analgesics (oral paracetamol 1000 mg, Ibuprofen 200 mg, or Dipyrone 1000 mg) at home, and refer to unit when these interventions were not helpful—no patient referred for this reason. All patients returned for a routine follow-up visit 2–3 weeks after surgery.

### Primary outcomes

The primary outcome was defined as the level of abdominal pain felt with change of position (according to the VAS scale) at 8 h after surgery. Based on previous studies^3,21^, it was estimated that each intervention (administration of SC and/or IP analgesia) would result in a 20% reduction in the primary outcome, as compared to non-intervention (administration of SC or IP placebo). In order to attain a power of 80% and a significance level of 0.05, the total sample size required was calculated to be 25 patients for each group. We included 30 patients in each group, in order to make up for any potential loss of follow up.

### Secondary outcomes

We measured the levels of pain experienced at rest (shoulder-tip and abdominal pain) at 3, 8, and 24 h after surgery. We also estimated the level of abdominal pain felt with change of position at 24 h after surgery.

Post-operative analgesic treatment was also measured, both in recovery and post-operative ward.

We estimated the amount of pain treatment given during recovery by calculating the MME given during recovery. The conversion factors for parenteral Morphine and Meperidine were 3 and 0.4 respectively^24^.

Additionally, we recorded prescriptions for analgesics in general and particularly opiates (Tramadol Hydrochloride) at 3, 8 and 24 h post-surgery.

### Statistical analyses

All statistical analyses were conducted using the R Statistical Software, version 3.5.2 (Foundation for Statistical Computing, Vienna, Austria)^25^.

### Univariate analyses

We compared the four study groups regarding patients' and surgery characteristics using Analysis of Variance (ANOVA) for continuous variables, and the Chi-square or Fisher exact tests for categorical variables.

The type of surgery was grouped into two categories: major (Hysterectomy, Myomectomy, and Endometrioma resection) and minor (Salpingectomy, Cystectomy, and Uni and Bilateral Salpingo-oophorectomy).

We compared the longitudinal outcomes (MME and discrete VAS scores) at each time point post-surgery) using ANOVA. Also, univariate mixed-effects linear models were conducted to estimate the univariate association between the study groups and those outcomes, using the surgeon's identity as a random effect.

We compared categorical outcomes (prescription for analgesics in general and for opiates at each time point post-surgery) using the Chi-square test. Also, we used univariate mix effects logistic models with the surgeon's identity as a random effect and calculated the univariate odds ratios for those outcomes.

### Multivariate analyses

We performed multivariate mixed-effects linear models to assess the associations between the study groups and MME during recovery and discrete VAS scores in each time point after surgery. The models were adjusted for the number of previous operations, BMI, the patient’s age, and the length and type (major vs. minor) of surgery, and were clustered for the surgeon's identity.

Multivariate mixed-effects logistic models were performed to assess the risk for the need for analgesic treatment at each time point after surgery, adjusting for the type of surgery (major vs. minor) and the MME given during recovery. Those models were also clustered for the surgeon's identity.

We also conducted secondary analyses, comparing observations with and without subcutaneous and intraperitoneal anesthesia. Those analyses were also performed using both linear and logistic mixed effects models.

### Statistical analyses

R Statistical Software, version 3.5.2, URL https://www.R-project.org.
